# Enhanced Iron Uptake in Plants by Volatile Emissions of *Rahnella aquatilis* JZ-GX1

**DOI:** 10.3389/fpls.2021.704000

**Published:** 2021-07-30

**Authors:** Wei-Liang Kong, Ya-Hui Wang, Xiao-Qin Wu

**Affiliations:** ^1^Co-Innovation Center for Sustainable Forestry in Southern China, College of Forestry, Nanjing Forestry University, Nanjing, China; ^2^Jiangsu Key Laboratory for Prevention and Management of Invasive Species, Nanjing Forestry University, Nanjing, China

**Keywords:** iron deficiency, *Rahnella aquatilis*, volatile organic compounds, ABA, *Arabidopsis thaliana*, *Medicago sativa*

## Abstract

Iron deficiency in soil has crucially restricted agricultural and forestry production. Volatile organic compounds (VOCs) produced by beneficial microorganisms have been proven to play an important role in inducing abiotic stress tolerance in plants. We investigated the effects of VOCs released by the rhizobacterium *Rahnella aquatilis* JZ-GX1 on the growth and root parameters of *Arabidopsis thaliana* under iron deficiency. The effect of the rhizobacterial VOCs on the gene expression in iron uptake and hormone signaling pathways were detected by RT-qPCR. Finally, the VOCs of the JZ-GX1 strain that could promote plant growth under iron deficiency stress were screened. The results showed that the JZ-GX1 strain could induce *A. thaliana* tolerance to iron deficiency stress by promoting the development of lateral roots and root hairs and increasing the activities of H^+^ ATPase and Fe^3+^ reductase. In addition, the *AHA2*, *FRO2*, and *IRT1* genes of *A. thaliana* exposed to JZ-GX1-emitted VOCs were upregulated 25-, 1. 81-, and 1.35-fold, respectively, and expression of the abscisic acid (ABA) synthesis gene *NCED3* was upregulated on both the 3rd and 5th days. Organic compounds were analyzed in the headspace of JZ-GX1 cultures, 2-undecanone and 3-methyl-1-butanol were found to promote *Medicago sativa* and *A. thaliana* growth under iron-limited conditions. These results demonstrated that the VOCs of *R. aquatilis* JZ-GX1 have good potential in promoting iron absorption in plants.

## Introduction

In contrast to animals, plants are fixed organisms, and their growth and development occur in the soil; thus, plants can suffer from a variety of biotic or abiotic stresses from the soil throughout their life cycle (Zhou et al., 2016a, 2019). As one of the essential micronutrients in plants, iron (Fe) plays a very important role in the electron transport chain and enzymatic reaction pathways in many physiological metabolic processes, such as photosynthesis, respiration, nitrogen fixation, protein, and nucleic acid synthesis (Zhou et al., 2016b). Although iron is abundant in most soils, its bioavailability is relatively low in alkaline or calcareous soils (Arikan et al., 2018). Iron forms extremely difficult-to-dissolve iron hydroxide, which is not easily utilized by plants (Freitas et al., 2015). It has been reported that calcareous soil covers approximately 1/3 of the Earth’s crust, and many plants growing on such soils often show chlorosis and yield loss related to iron deficiency (Tsai and Schmidt, 2017).

To cope with iron deficiency, non-gramineous monocotyledons and dicotyledons mainly use reduction-based strategies (strategy I), while gramineous monocotyledons use chelation-based strategies (strategy II), although it is known that there are plants that can use both (Flores-Cortez et al., 2019). In strategy I, iron uptake by plants consists of three steps: (1) release of protons into the plant rhizosphere to reduce pH and increase the solubility of insoluble iron oxide by H^+^ ATPase (*AHA2*), (2) reduction of Fe^3+^ to ferrous form by plasma membrane-bound chelating iron reductase (*FRO2*), and (3) transport of reduced Fe^2+^ to the root epidermis through divalent metal transport protein IRT1 across the plasma membrane (Del Carmen Orozco-Mosqueda et al., 2013). *FRO2* and *IRT1* are finely regulated by iron deficiency-induced transcription factors (*FIT1*), and *FIT1* plays a central role in iron homeostasis (Montejano-Ramirez et al., 2018). However, neither gramineous nor non-gramineous monocotyledons can obtain enough iron in alkaline or calcareous soils by strategy I or strategy II (Castulo-Rubio et al., 2015). Thus, for the sustainable development of agriculture, there is an urgent need to improve the ability of plants to absorb iron from low-iron available soils.

The use of plant growth promoting rhizobacteria (PGPR) to help plants obtain available iron is considered to be environmentally friendly (Masalha et al., 2000; Rroco and Kosegarten, 2003; Montejano-Ramirez et al., 2015). It is known that several beneficial microorganisms can promote iron uptake by plants based on the mechanisms of chelation, reduction, acidification and induction, among which the induction of plant systemic resistance mediated by volatile organic compounds (VOCs) has attracted wide attention in recent years (Back et al., 2020). Compared with soluble compounds, the VOCs released by rhizosphere microorganisms have two advantages in the interaction of microbial communities: first, volatile compounds with low molecular weights (<300 Da) can evaporate and migrate freely via soil pores over long distances (Sharifi and Ryu, 2018a,b); second, these gaseous substances act as signal molecules and can activate or enhance plant defense responses (Fincheira and Quiroz, 2018; Netzker et al., 2020). Some studies showed that *Bacillus subtilis* GB03 directly and indirectly promoted the growth of *Arabidopsis thaliana* under iron deficiency through the emission of acetoin and 2,3-butanediol (Zhang et al., 2009). *Arthrobacter agilis* UMCV2 induced iron acquisition in *Medicago sativa in vitro* via dimethyl hexadecylamine (Elizabeth Aviles-Garcia et al., 2016; Ramirez-Ordorica et al., 2020). The airborne signals from *Trichoderma asperellum* T-34 increased the expression of the iron uptake genes *LeFER*, *LeFRO*, and *LeIRT* in tomato roots (Martinez-Medina et al., 2017). The transcription factor MYB72, which is related to induced systemic resistance (ISR) and iron uptake in *A. thaliana* roots, is activated by VOCs released by *Pseudomonas simiae* WCS417 and enhances the iron acquisition and systemic immunity of *A. thaliana* roots at the same time (Zamioudis et al., 2015).

A previous study by our laboratory showed that *Rahnella aquatilis* JZ-GX1 could promote iron absorption in *Cinnamomum camphora* by producing siderophores and organic acids, thus alleviating iron deficiency-induced chlorosis (Kong et al., 2020b). However, it is not clear whether the tested strain can further induce the systemic tolerance of plants to iron deficiency stress by producing VOCs. For this reason, in this study, *A. thaliana* and *M. sativa* model plants were used in a two-grid Petri dish system to: (1) explore the effects of VOCs produced by *R. aquatilis* JZ-GX1 on the growth of *A. thaliana* under iron-deficient conditions; (2) clarify the signaling pathway of resistance to iron deficiency stress induced by the JZ-GX1 strain in *A. thaliana*; and (3) identify one or more VOCs produced by the JZ-GX1 strain that act as elicitors.

## Materials and Methods

### Plant Preparation

In this study, we used *A. thaliana* wild-type accession Col-0 and *M. sativa* as our test subjects. First, the seeds of these two plants were treated with 70% ethanol for 5 min and 2.6% NaClO for 1 min and then rinsed with sterile water 7–8 times for surface sterilization. The seeds were poured into 1/2 Murashige and Skoog (MS) agar-solidified medium with sterile water; a pipette was used to remove the sterile water, and the seeds were spread evenly and as far apart as possible. The Petri dish was placed on a super-clean table and blown dry with a fan, sealed with parafilm, and vernalized in a refrigerator at 4°C for 2 days. The Petri dish was placed vertically in a light incubator for 5 days to raise seedlings. The conditions of the light incubator were as follows: 16 h of light, 8 h of dark, 70% relative humidity, a light intensity of 4000 lux and a temperature of 25°C (Zhou et al., 2017).

### Bacterial Cultures

*Rahnella aquatilis* JZ-GX1 is a plant growth-promoting bacterium isolated from the rhizosphere soil of a 28-year-old *Pinus massoniana* in Nanning, Guangxi. It is now stored in the typical Culture Preservation Center of China (CCTCC, NO: M2012439). This strain was inoculated on Luria-Bertani (LB) medium for activation, and then a single colony was selected and transferred into a shake flask containing LB liquid medium and then incubated at 28°C and 180 rpm. The bacterial liquid was diluted to 1 × 10^7^ CFU/mL with phosphate buffer at pH 7.8.

### Co-culture of Plants and Bacteria

To study the effect of VOCs released by the JZ-GX1 strain on the growth of *A. thaliana* under iron-limited conditions, I-plates (90 mm in diameter) were used in this experiment. Each petri dish was divided into two chambers such that the non-gaseous metabolites produced by bacteria could not reach *A. thaliana*, which was inoculated in a separate chamber; thus, the bacteria could only affect *A. thaliana* through VOC signals over the plate. One chamber of the Petri dish contained 1/2 MS medium, and the other contained LB medium. 1 M KOH (containing 6 mM NaHCO_3_) was added to the 1/2 MS medium on which the *A. thaliana* side was placed to a final pH of 8.0 as iron deficiency treatment. Seven-day-old *A. thaliana* seedlings were transplanted into the chamber containing 1/2 MS medium, five seedlings were transplanted into each dish, and the distance between seedlings was the same. Seedlings with good and consistent growth were selected when transplanting. The hypocotyl part of the seedling was gently clamped with sterilized tweezers and then transplanted into the upper part of the Petri dish. 10 μL of bacterial solution was added to the other compartment, and LB medium without bacterial solution was used as the control. After the bacterial solution was homogeneously distributed in the culture medium, the Petri dish was sealed with parafilm and cultured vertically in a light incubator for 14 days (Wang et al., 2017). The culture conditions were the same as described in plant preparation.

### Determination of Plant Root Parameters

After the bacterium was co-cultured with *A. thaliana* for 14 days, the Petri dish was removed to observe the overall effect of VOCs produced by the bacterium on *A. thaliana*, and the root tips of *A. thaliana* were photographed with a Zeiss stereomicroscope (Zeiss Microscope System Standard 16; Carl Zeiss Ltd., Germany). Measurements were determined using Image J Tool software; pixel areas were calibrated based on a known distance. Root hairs length were measured in a region of 500 μm at approximately 1 cm from the primary root tip. The *A. thaliana* seedlings were removed and weighed with a 1/10000 balance; the length of the main root of *A. thaliana* was measured and recorded with a Vernier caliper. Then, the seedlings were placed in a Petri dish filled with clear water so that the roots could be fully elongated, and the number of lateral roots was counted and recorded (Perez-Flores et al., 2017).

### Leaf Chlorophyll

Leaves (500 mg) were combined with a small amount of 80% acetone and quartz sand were then ground into a homogenate. Then, additional 80% acetone was added, the homogenate was transferred to a centrifuge tube, the volume was brought to 10 mL, and the tube was centrifuged at 12000 *g* and 4°C for 15 min. The absorbance of the supernatant was detected at 645 and 663 nm, and the total chlorophyll content was calculated as follows: total chlorophyll = (8.02A663 + 20.21A645) × V/1000 × W, where V is the total extract volume and W is the sample weight (Castulo-Rubio et al., 2015).

### Determination of Endogenous Iron Content

The leaves from the seedlings were chopped and placed in 1 mol/L HCl at a 1:10 ratio (v/v) for 24 h (Koseoglu and Acikgoz, 1995). The extract was filtered and assayed using an atomic absorption spectrophotometer (AA900T, Perkin Elmer).

### Visualization of Rhizosphere Proton Release

The change in pH value in the rhizosphere was determined by the staining location method. A total of 1 g of agar, 13.6 mg of CaSO_4_ and 6 mg of bromocresol violet were added to 100 mL of distilled water, heated to dissolve, mixed well, and cooled to approximately 50°C, and the pH was adjusted until the solution color was dark red. The liquid on the root surface was drained, the roots were placed flat on the bottom of a culture dish, and the prepared agar containing the indicator was evenly and quickly poured on the culture dish at a thickness of approximately 0.8–1 cm. After agar solidification, the dish was placed in the dark at room temperature for 24 h, and the change in indicator color was observed (Li et al., 2020).

### Quantitative Real-Time PCR

Seven-day-old *A. thaliana* seedlings were co-cultured with bacterial VOCs for 3 and 5 days, frozen with liquid nitrogen and fully ground, and the total RNA of *A. thaliana* was extracted by a rapid plant RNA extraction kit (Beijing Zoman Biotechnology Co., Ltd., Beijing, China). The relative expression levels of the *AHA2*, *FRO2*, *IRT1*, *FIT*, *PDF1*, *NPR1*, *ERF1*, and *NCED3* genes were determined, and the *Actin* gene was used as the internal control (Zhou et al., 2016c; Ines Dinolfo et al., 2017). The expression levels of related genes were calculated by ABI 7500 software (Applied Biosystems, United States) and the 2^–ΔΔCT^ method (Kong et al., 2020a). The primers used to amplify these genes are listed in Table 1.

**TABLE 1:** Primers used in RT-qPCR analysis.

**Gene name**	**Gene function**	**Primers**
*AHA2*	Plasma membrane H^+^ ATPase	GAGAATGTGCATGTGCCAAA
		TGACTGATCTTCGATCCTCTCA
*FRO2*	Fe^3+^ reductase	TGCCACAAAGATTCGTCATGTGCG
		TGTGGCTCTTCTTCTCTGGTGCTT
*IRT1*	Fe^2+^ transporter	TCCCGGAGGCGAAACACTTAATGA
		ACCCGTGCGTCAACAAAGCTAAAG
*FIT*	Iron deficiency-induced transcription factors	TTCATCTTCTTCACCACCGGCTCT
		ACCTCTTCGACGAATTGCCTGACT
*PDF1*	Jasmonic acid biosynthesis	CAACGGGAAAATAAACATTAAAACAG
		CTGTTACGTCCCATGTTAAATCTACC
*NPR1*	Salicylic acid biosynthesis	AACCGTGGAACTCGGGAAACGA
		GTCTTCTCCGCAAGCCAGTTGA
*ERF1*	Ethylene biosynthesis	TCCCGAGCCAAACCCTAATAC
		CCTTCCGATCAAATCCGTAAG
*NCED3*	Abscisic acid biosynthesis	TTAGCTCCGTTGCGCACATA
		ATCTGCGCTTCACACTCCTC
*YUC1*	Auxin biosynthesis	CAAAGAAAGGAGCAAAGTTTATGG
		CTGAAGCCAAGTAGGCACGTT
*YUC8*	Auxin biosynthesis	TGTATGCGGTTGGGTTTACG
		CAGAGCCTATGTCTTGTGCGAT
*Actin*	Endogenous control, Reference gene	GAAATCACAGCACTTGCA
		AGCCTTTGATCTTGAGAG
*MsAHA2*	Plasma membrane H^+^ ATPase	TCATGGGTCATGGAAATG
		CTCCTGGGACAAGAATAGC
*MsIRT1*	Fe^2+^ transporter	CCCTAGCTGATTGTGAAAGT
		TCCCAAGAATAATACCAGCC
*MsBHLH1*	Iron deficiency-induced transcription factors	TTATCCTTCATTCGGCTTCG
		TGATCCTACTTCTTCACTTGGTTC
*MsActin*	Endogenous control, Reference gene	CTCTCAAGTACCCCATTGAGC
		TATTGGCCTTTGGGTTAAGTG

### Determination of H^+^ ATPase and Fe^3+^ Reductase Activities in Plants

To verify whether the expression of key iron absorption genes was consistent with enzyme activity, H^+^ ATPase and Fe^3+^ reductase were extracted from plant roots according to the research of Zhang et al. (2009) and Arikan et al. (2018) and their activities were measured at 450 and 562 nm, respectively.

### Verification of the Selected Synthetic VOCs on the Plant Growth Under Iron-Limited Medium

According to our previous research (Kong et al., 2020a), the authentic reference standard compounds detected by GC-MS were diluted with dimethyl sulfoxide to 10, 50, 100, and 200 μM and were added to one side of the Petri dish; no compounds were added to the Petri dish in the control test (Pinedo et al., 2015; Ledger et al., 2016). The plate was cultured vertically in a light incubator, and each compound at each concentration was tested as four parallel samples. The culture conditions were the same as detailed above. After 14 days of culture, the results were observed, and the growth indexes of the plants were determined.

### Data Analysis and Processing

The data were subjected to analysis of variance and Duncan’s multiple comparison test with SPSS 21.0 software (IBM Inc., Armonk, NY, United States), and the standard errors of all mean values were calculated (*p* < 0.05). Graphs were generated using GraphPad Prism 8.0 (GraphPad Software, Inc., United States).

## Results

### *R. aquatilis* JZ-GX1 Promoted the Growth of *A. thaliana* Under Iron Deficiency Through the Emission of VOCs

**FIGURE 1:**
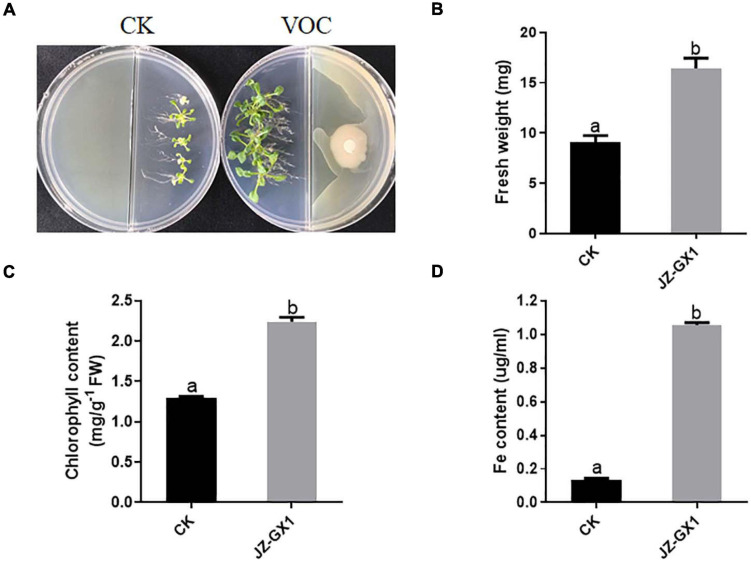
Effects of VOCs of *Rahnella aquatilis* JZ-GX1 on the growth of *Arabidopsis thaliana* under iron limitation. **(A)** Plant phenotype, **(B)** plant fresh weight, **(C)** chlorophyll content, and **(D)** active iron content. CK: control. One-way ANOVA was performed, and Duncan’s *post hoc* test was applied. Different letters indicate statistically significant differences (*p* < 0.05) among treatments.

*Arabidopsis thaliana* was cultured with 1/2 MS medium (pH = 8.0) on one side of a Petri dish, and the tested bacterium was cultured on the other side with LB medium. After 14 days, the interaction results were observed, and the fresh weight and chlorophyll and active iron contents of the plants were determined. The growth-promoting effect of the JZ-GX1 strain on *A. thaliana* was analyzed. These results showed that the growth of 14-day-old *A. thaliana* in an alkaline environment was significantly inhibited; the plants were thin and the leaves were yellowish, while the *A. thaliana* plants co-cultured with the JZ-GX1 strain under iron-limited conditions were healthy and had dark green leaves (Figure 1A); the fresh weight, chlorophyll content and active iron content of the co-cultured plants increased by 2.71%, 58.11%, and 7.84 times, respectively (Figures 1B–D).

### *R. aquatilis* JZ-GX1 VOCs Changed the Root System Architecture of *A. thaliana* Under Iron Deficiency Stress

**FIGURE 2:**
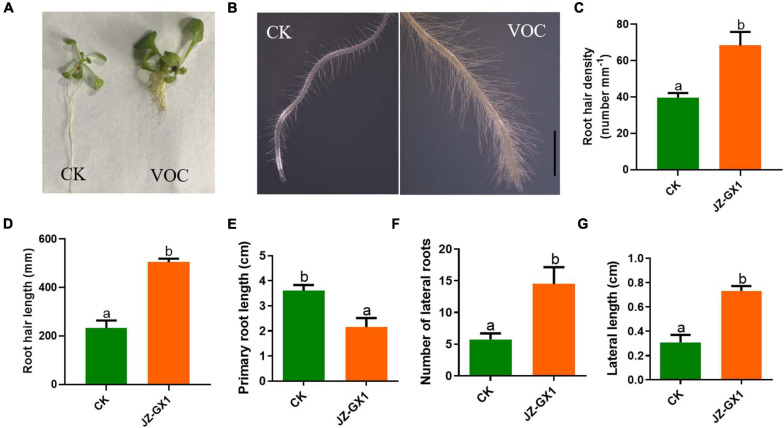
Effect of *Rahnella aquatilis* JZ-GX1 VOCs on the root system architecture of *Arabidopsis thaliana* compared to the control (CK) under iron deficiency stress. **(A)** The overall structure of the root system, **(B)** root hair morphology, **(C)** root hair density, **(D)** root hair length, **(E)** primary root length, **(F)** lateral root number, and **(G)** lateral root length. The scale is 0.5 mm. One-way ANOVA was performed, and Duncan’s post hoc test was applied. Different letters indicate statistically significant differences (*p* <0.05) among treatments.

The development of the root system determines the ability of plants to absorb water and nutrients. In this experiment, the effects of VOCs produced by the tested strains on root hair production, primary root length, lateral root length, and number of lateral roots in *A. thaliana* were studied. From Figures 2A,E, it was observed that the elongation of the main root of *A. thaliana* seedlings exposed to JZ-GX1 VOCs was significantly inhibited, while the number of lateral roots increased by 2.53 times compared with that of the control (Figure 2F). At the same time, the length of the lateral root of the control was 0.309 cm, while that of *A. thaliana* treated with JZ-GX1 VOCs was 0.732 cm, which increased by 2.37 times (Figure 2G). Using stereomicroscopy, it was observed that the root hairs of *Arabidopsis* seedlings treated with VOCs were more developed and denser than those of the control seedlings (Figures 2B–D).

### *R. aquatilis* JZ-GX1 VOCs Regulate the Expression of Genes Related to Iron Uptake in *A. thaliana*

**FIGURE 3:**
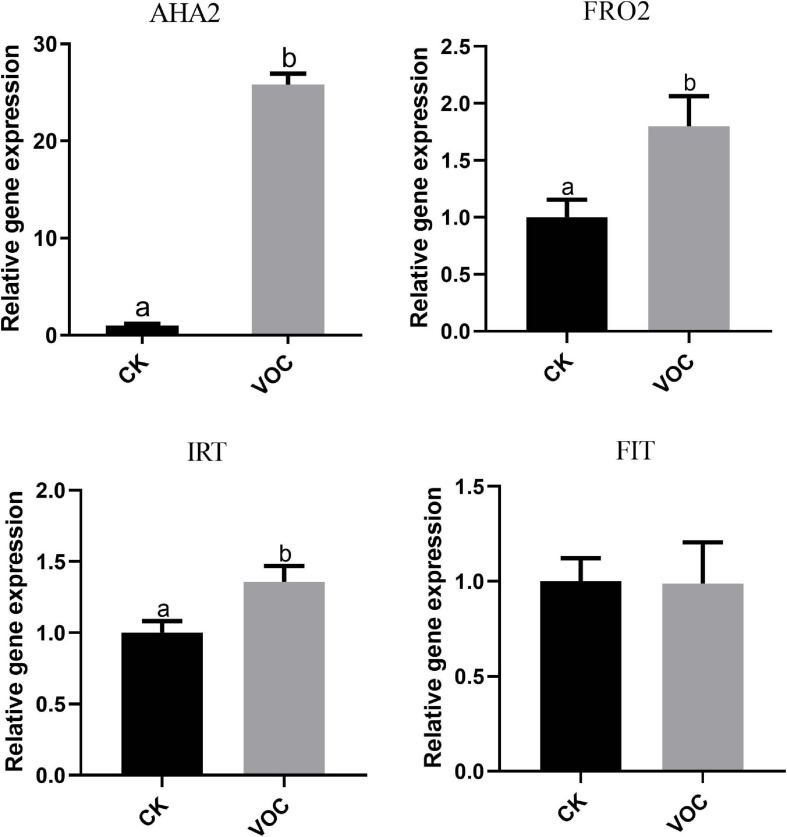
Effects of VOCs from *Rahnella aquatilis* JZ-GX1 on the expression of the *AHA2*, *FRO2*, *IRT1*, and *FIT* genes in *Arabidopsis thaliana.* One-way ANOVA was performed, and Duncan’s *post hoc* test was applied. Different letters indicate statistically significant differences (*p* < 0.05) among treatments.

To determine whether JZ-GX1 can regulate Fe^3+^ reductase and Fe^2+^ transporter genes in plants, we analyzed the transcript abundance of H^+^ ATPase (*AHA2*), Fe^3+^ reductase (*FRO2*), and Fe^2+^ transporter (*IRT1*) genes in *A. thaliana* grown in iron-deficient medium. Compared with that in the control plants, the expression of *AHA2*, *FRO2*, and *IRT1* in the plants exposed to JZ-GX1 VOCs for 4 days was significantly upregulated and was 25, 1.81, and 1.357 times higher than that in the control plants, respectively. However, there was no significant difference in the iron transcriptional regulator *FIT* (Figure 3). The abundance and activity of iron-obtained transcripts increased in plants treated with VOCs produced by the JZ-GX1 strain, which indicated that the JZ-GX1 strain activated iron acquisition in plants by regulating the strategy I plant iron deficiency response.

### *R. aquatilis* JZ-GX1 VOCs Activated Iron Absorption in *A. thaliana*

**FIGURE 4:**
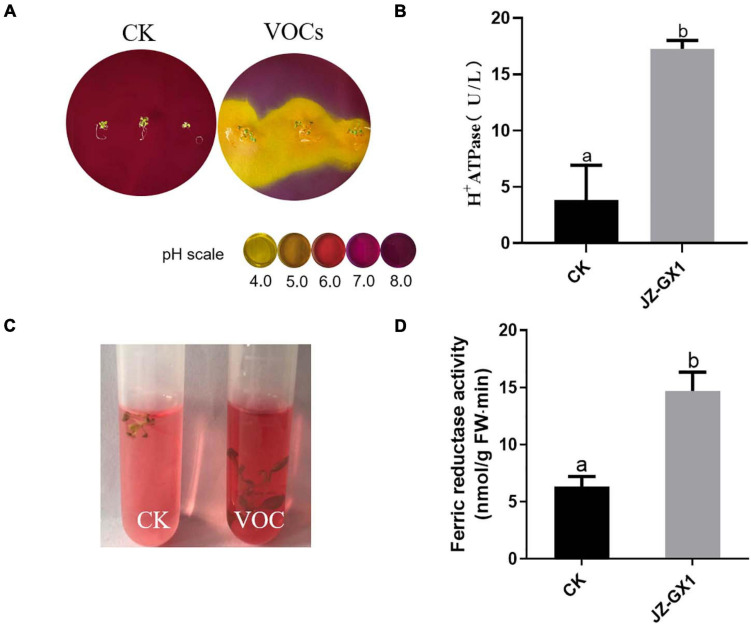
Effects of VOCs from *Rahnella aquatilis* JZ-GX1 on H^+^ ATPase and ferrate reductase activities in *Arabidopsis thaliana*. **(A)** H^+^ secretion, **(B)** H^+^ ATPase enzyme activity, **(C)** ferrate reductase reaction, and **(D)** quantitative analysis. CK: control. One-way ANOVA was performed, and Duncan’s *post hoc* test was applied. Different letters indicate statistically significant differences (*p* < 0.05) among treatments.

H^+^ ATPase and ferrate reductase are key enzymes in the process of iron uptake by plants. To test whether the JZ-GX1 strain affected the ability of plants to acidify the rhizosphere, bromocresol purple, an indicator of pH, was added to the medium for chromogenic acidification. Plants were initially grown in iron-deficient media for 14 days (including treatments exposed to JZ-GX1 and control treatments) and then transferred to the medium containing pH indicator, which turned yellow when the pH decreased to below 5.0. The results showed that within 24 h of transfer to the indicator medium, plants exposed to JZ-GX1 showed significant rhizosphere acidification compared with the control plants (Figure 4A). Accordingly, the enzyme activity of H^+^ ATPase was 4.51 times higher than that of the control (Figure 4B). In addition, the iron reductase activity of the plants exposed to JZ-GX1 VOCs was 2.32 times higher than that of the control (Figures 4C,D). The above experiments show that the JZ-GX1 strain can not only effectively reduce the pH value of the rhizosphere by inducing root proton release but also trigger *Arabidopsis* roots to secrete more ferrate reductase, thus transforming insoluble Fe^3+^ into Fe^2+^ for plant absorption and utilization.

### Changes in the Iron Deficiency Resistance Pathway in *A. thaliana* Induced by *R. aquatilis* JZ-GX1 VOCs

**FIGURE 5:**
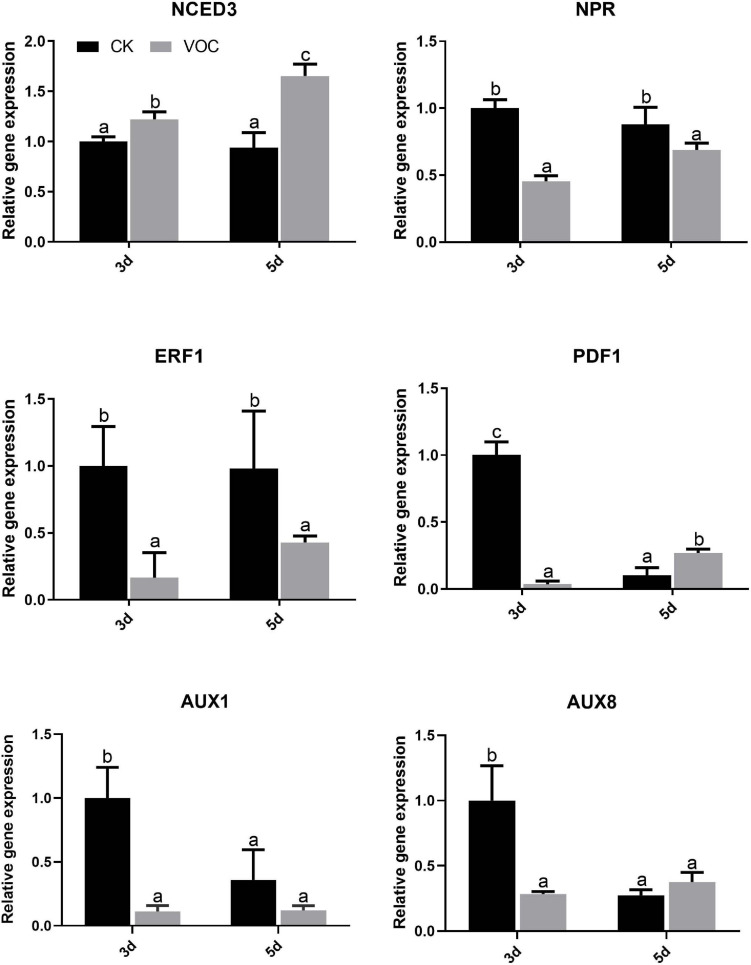
Effects of VOCs from *Rahnella aquatilis* JZ-GX1 on the transcriptional expression of genes related to hormone biosynthesis in *Arabidopsis thaliana.* One-way ANOVA was performed, and Duncan’s *post hoc* test was applied. Different letters indicate statistically significant differences (*p* < 0.05) among treatments.

To explore the signaling pathway of *A. thaliana* tolerance to low iron induced by the JZ-GX1 strain, the key genes in the biosynthesis pathways of jasmonic acid (JA), salicylic acid (SA), ethylene (ET), auxin, and abscisic acid (ABA) were detected by qPCR. Compared with that in the control plants, the expression of *NCED3* in the ABA signaling pathway of the plants treated with JZ-GX1 was upregulated 3 days after exposure to VOCs and was approximately 1.223 times higher than that in the control plants; this pathway was still upregulated on the 5th day, and the expression was approximately 1.654 times higher than that of the control plants. However, the expression of *NPR* in the SA signaling pathway, *ERF1* in the ET pathway, *PDF1* in the JA signaling pathway and YUC1, YUC8 in the auxin signaling pathway were downregulated on the third and 5th day (Figure 5). It was suggested that the VOCs produced by the JZ-GX1 strain induced *A. thaliana* resistance to iron deficiency stress through the ABA-mediated signaling pathway.

### *R. aquatilis* JZ-GX1 Promoted the Growth of *M. sativa* Under Iron Deficiency

**FIGURE 6:**
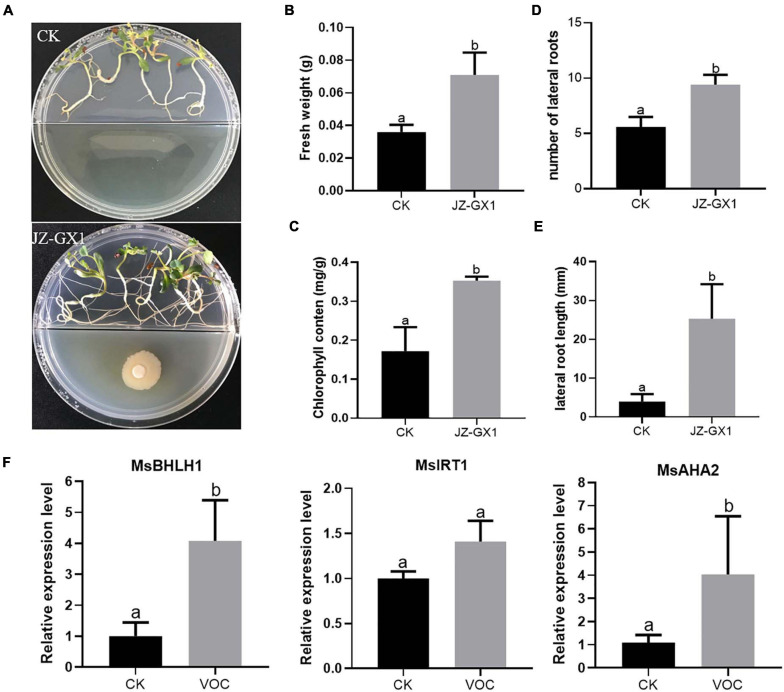
Effect of VOCs from *Rahnella aquatilis* JZ-GX1 on the growth of *Medicago sativa* under iron-deficient conditions. **(A)** Plant phenotype, **(B)** fresh weight, **(C)** chlorophyll content, **(D)** lateral root number, **(E)** lateral root length, and **(F)** relative expression of *MsBHLH1*, *MsIRT1*, and *MsAHA2* in roots of *M. sativa* seedlings that were CK (control) or treated with VOCs from *R. aquatilis* JZ-GX1 (VOC). One-way ANOVA was performed, and Duncan’s *post hoc* test was applied. Different letters indicate statistically significant differences (*p* < 0.05) among treatments.

To investigate whether we could reproduce the effect of bacterial VOCs also in other plant species than *Arabidopsis*, we investigated the impact of JZ-GX1 VOCs on the Fe deficiency response in *M. sativa* seedlings. At 14 days, *M. sativa* exposed to VOCs of the JZ-GX1 strain showed better development and growth than the control plants (Figure 6A), resulting in a significant increase in plant fresh weight and chlorophyll content (Figures 6B,C). The root system of the seedlings treated with bacterial VOCs was more developed than that of untreated control seedlings, and the number and length of lateral roots increased by 1.68 and 6.41 times, respectively (Figures 6D,E). In addition, bacterial VOCs up-regulated the expression of *MsBHLH1*, *MsAHA2*, and *MsIRT1* (Figure 6F). These results showed a very clear promotional effect is demonstrated both in *A. thaliana* and *M. sativa*.

### Effect of Specific VOCs Released by *R. aquatilis* JZ-GX1 on the Growth of *M. sativa* Under Iron-Deficient Conditions

**FIGURE 7:**
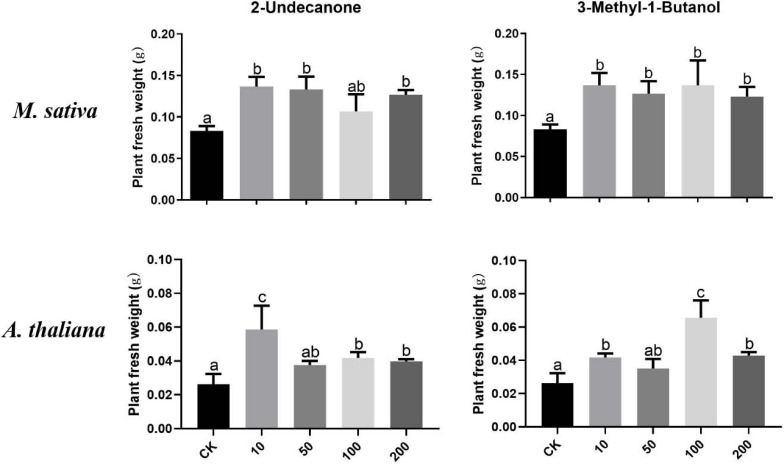
Effects of pure compounds at different concentrations on the growth of *M. sativa* and *A. thaliana* under iron deficiency. CK: control. One-way ANOVA was performed, and Duncan’s *post hoc* test was applied. Different letters indicate statistically significant differences (*p* < 0.05) among treatments.

According to the VOC profile of the JZ-GX1 strain obtained from previous experiments (Kong et al., 2020a), we tested the growth-promoting effects of eight VOCs on plants. Figure 7 shows that 2-undecanone and 3-methyl-1-butanol could promote the growth of *M. sativa* under iron deficiency stress. When 10–200 μM 2-undecanone solutions were added, the fresh weight of *M. sativa* increased, and there is no significant difference among different concentrations (*p* < 0.05). Similarly, these two compounds significantly promoted the growth of *A. thaliana* under iron deficiency. The plant fresh weight reached the maximum when a small amount (10 μM) of 2-undecanone was added, and when 100 μM of 3-methyl-1-butanol was added, the effect was the best (*p* < 0.05). The six other compounds had no significant effect on plant growth.

## Discussion

To maintain normal growth and development, most plants form robust roots to obtain water and mineral nutrients from the soil (Dahmani et al., 2020). Root system architecture (RSA) integrates the topological structure of the root system, the spatial distribution of the main root and lateral roots, and the number and length of various types of root systems (Thanh et al., 2020). Some abiotic and biological factors, including plant growth-promoting rhizobacteria (PGPR), can affect the RSA. The most common effect of PGPR is to inhibit the growth of primary roots, increase the proliferation of lateral roots and root hairs, and lead to an increase in aboveground biomass. The other effect is that the increase in plant biomass is accompanied by an increase in primary root growth (Zhou et al., 2016a; Sun et al., 2020). In this study, the VOCs of *R. aquatilis* JZ-GX1 increased the length and number of lateral roots in *A. thaliana* and *M. sativa* under iron deficiency stress, which not only increased the distribution sites of Fe^3+^ reductase in roots but also increased the area of iron nutrition absorbed by roots. Accordingly, these observations suggest that the VOCs released by *R. aquatilis* JZ-GX1 enhance the capacity of the host root to access Fe by modulating morphological adaptive responses to iron-deficient conditions.

Rhizosphere acidification is very important for plants to absorb iron. Studies have shown that when the soil pH increases by 1.0, the solubility of iron decreases 1000 times (Zhou et al., 2018). Previous researchers used MS medium to remove iron salt when studying the effect of microbial volatiles on the growth of *A. thaliana* under iron deficiency (Castulo-Rubio et al., 2015; Wang et al., 2017; Montejano-Ramirez et al., 2018), but in this experiment, the total nutrient MS medium pH was directly adjusted to 8.0. The growth environment of *A. thaliana* was relatively close to environmentally relevant alkaline soil conditions; that is, the soil was not free of iron, but the high-pH environment led to a decrease in iron availability (Pii et al., 2015; Aras et al., 2018). In the dichotomous dish culture experiment, the roots of *A. thaliana* seedlings treated with JZ-GX1 VOCs changed from purple to yellow, indicating that the acidification ability of *A. thaliana* seedlings was significantly enhanced, and the significant increase in H^+^ ATPase activity in *A. thaliana* seedlings treated with JZ-GX1 VOCs could explain this acidification effect. Thus, the rapid decrease in pH value was beneficial to the dissolution of iron in the rhizosphere. Second, the *Arabidopsis* root iron reductase gene was highly expressed, and a higher iron reductase activity was detected in *A. thaliana* roots exposed to JZ-GX1 VOCs than in control seedling roots. Studies have shown that the optimum pH environment for Fe^3+^ reductase is 5.6 (Arikan et al., 2018). Although plants in alkaline soils initiate their own adaptive response to iron deficiency stress, they are quickly buffered by high pH values, which explains why some iron deficiency-sensitive plants are prone to yellowing in calcareous soils (Zhou et al., 2016b). Therefore, the VOCs released by *R. aquatilis* JZ-GX1 can enhance the ability of *A. thaliana* to absorb iron by activating the physiological response of the plant itself.

Although it has been reported that microorganisms induce plant iron uptake by releasing VOCs, there are few studies on the related signaling pathways. It is well known that the phytohormones auxin, ET, JA, and SA are involved in the regulation of resistance to abiotic stresses. But it seems that this four pathways had no effect on the induction of iron deficiency in plants conferred by JZ-GX1 VOCs, because JZ-GX1 VOCs significantly suppressed the expression of *YUC1*, *YUC8*, *NPR*, *ERF1*, *PDF1* that involved in auxin, ET, JA, and SA synthesis in plants under iron deficiency condition. Only *NCED3* in the ABA synthesis pathway was induced and expressed in large quantities on the 3rd and 5th days. *NCED3* is an enzyme that encodes ABA biosynthesis, namely, 9-cis-epoxide dioxygenase (Xing et al., 2019). Zhang et al. (2020) reported that the addition of exogenous ABA can alleviate iron deficiency in apples and promote long-distance iron transport in plants by regulating the distribution of iron in roots and stems. Under iron-deficient conditions, exogenous ABA can also promote the activation of apoplast iron by promoting an increase in phenols secreted by *Arabidopsis* roots and lead to an increase in citric acid concentration and iron concentration in xylem sap, thus increasing the available iron content in the aboveground parts of plants (Lei et al., 2014). In addition, ABA can promote the hydrolytic activity and proton transport of H^+^ ATPase (Olaetxea et al., 2019). In our experimental results, the expression of *AHA2* in *A. thaliana* exposed to JZ-GX1 VOCs for 5 days was upregulated 25-fold, which may be due to the increase in endogenous ABA content in *A. thaliana*. However, different results were obtained in other studies. Liu et al. (2020b) found that in addition to ABA signaling, JA signaling also played important roles in mediating systemic salt stress tolerance after inoculating FZB42 on the roots. Chen et al. (2016) found that the expression of NCED was down-regulated with the inoculation of SQR9 under salt stress condition. In addition, Bhattacharyya et al. (2015) showed that VOCs emitted by *Alcaligenes faecalis* JBCS1294 induced salt tolerance in *Arabidopsis* by modulating the auxin and gibberellin pathways. These different results might be explained by different stress environments of plants or differences in chemical signaling by PGPR VOCs.

To identify the compounds involved in the regulation of plant iron absorption in the VOCs of *R. aquatilis* JZ-GX1, we analyzed the present VOCs by gas chromatography-mass spectrometry in a preliminary experiment (Kong et al., 2020a). In this study, after concentration screening, we finally identified two bioactive substances among the eight VOCs obtained. The addition of low concentrations of 2-undecanone and 3-methyl-1-butanol could improve the growth of *M. sativa* and *A. thaliana* under iron deficiency stress. In one study, these two substances were reported to be produced by *Paraburkholderia phytofirmans* PsJN and could also induce the growth of *A. thaliana* under salt stress (Ledger et al., 2016). The plant fresh weight measured in this study were significantly increased with exposure to these two compounds, but the enhancement effect was not as good as that of the JZ-GX1 strain treatment group. This is a common phenomenon observed in other studies (Liu et al., 2020b); that is, compared with commercially available, pure 2-undecanone or 3-methyl-1-butanol, the mixture of JZ-GX1 VOCs was more effective. This can be explained by the concentration of these two substances in the VOC mixture. In the plant-bacteria interaction system, the concentration of 2-undecanone or 3-methyl-1-butanol was unknown, so we could not determine the true concentration of these two substances after the interaction between bacteria and plants. Another possible explanation is that the JZ-GX1 strain produced VOCs other than 2-undecanone or 3-methyl-1-butanol and that one or more of these VOCs contributed to stress tolerance induction, or there are probably other volatile compounds that work in synergy and were not detected.

Plant growth promoting rhizobacteria have great potential in helping plants cope with adversity (Pinedo et al., 2015; Yang et al., 2020). An increasing number of studies have realized that microbial VOCs play an important ecological role in mediating interspecies and intraspecific interactions (Vaishnav et al., 2016; Del Rosario Cappellari and Banchio, 2020; Rivera-Mendez et al., 2020). In this study, it is reported for the first time that VOCs produced by *R. aquatilis* can promote plant growth under iron-deficient conditions. In view of the fact that salt stress and alkali stress occur simultaneously under natural conditions (Liu et al., 2020b; Liang and Shi, 2021), it is necessary to explore whether VOCs from the JZ-GX1 strain can promote plant growth under salt stress. Second, the molecular mechanism of how plants sense gas signals emitted by bacteria should be further revealed.

## Data Availability Statement

The raw data supporting the conclusions of this article will be made available by the authors, without undue reservation.

## Author Contributions

W-LK completed the experimental research and the first draft of the manuscript. Y-HW participated in the experimental result analysis. X-QW directed the experimental design, data analysis, and manuscript writing and revision. All authors read and agreed on the final text.

## Conflict of Interest

The authors declare that the research was conducted in the absence of any commercial or financial relationships that could be construed as a potential conflict of interest.

## Publisher’s Note

All claims expressed in this article are solely those of the authors and do not necessarily represent those of their affiliated organizations, or those of the publisher, the editors and the reviewers. Any product that may be evaluated in this article, or claim that may be made by its manufacturer, is not guaranteed or endorsed by the publisher.
